# Intrabiliary and abdominal rupture of hepatic hydatid cyst leading to biliary obstruction, cholangitis, pancreatitis, peritonitis and septicemia: a case report

**DOI:** 10.1186/s13256-021-02822-5

**Published:** 2021-05-28

**Authors:** Manouchehr Aghajanzadeh, Mohammad Taghi Ashoobi, Hossein Hemmati, Pirooz Samidoust, Mohammad Sadegh Esmaeili Delshad, Yousha Pourahmadi

**Affiliations:** 1 Inflammatory Lung Disease Research Center, Razi Hospital, Guilan University of Medical Sciences, Rasht, Iran; 2 Razi Clinical Research Development Unit, Razi Hospital, Guilan University of Medical Sciences, Rasht, Iran

**Keywords:** Hydatid cyst, Liver, Intrabiliary rupture

## Abstract

**Background:**

Hydatid cysts are fluid-filled sacs containing immature forms of parastic tapeworms of the genus *Echinococcus*. The most prevalent and serious complication of hydatid disease is intrabiliary rupture, also known as cystobiliary fistulae. In this study, a sporadic case of biliary obstruction, cholangitis, and septicemia is described secondary to hydatid cyst rupture into the common bile duct and intraperitoneal cavity.

**Case presentation:**

A 21-year-old Iranian man was admitted to the emergency ward with 5 days of serious sickness and a history of right upper quadrant abdominal pain, fatigue, fever, icterus, vomiting, and no appetite. In the physical examination, abdominal tenderness was detected in all four quadrants and in the scleral icterus. Abdominal ultrasound revealed intrahepatic and extrahepatic biliary duct dilation. Gallbladder wall thickening was normal but was very dilated, and large unilocular intact hepatic cysts were detected in segment IV and another one segment II which had detached laminated membranes and was a ruptured or complicated liver cyst.

**Conclusion:**

Intrabiliary perforation of the liver hydatid cyst is an infrequent event but has severe consequences. Therefore, when patients complain of abdominal pain, fever, peritonitis, decreased appetite, and jaundice, a differential diagnosis of hydatid disease needs to be taken into consideration. Early diagnosis of complications and aggressive treatments, such as endoscopic retrograde cholangiopancreatography and surgery, are vital.

## Background

Hydatid disease, also known as echinococcosis, is a serious and problematic health issue worldwide, and this parasitic disease is widespread in most Mediterranean countries, West Asia, South America, the Far East, Australia, and East Africa [1–3]. Humans are intermediate and incidental hosts and are infected either through the direct route via exposure to or contact with infected dogs or other canines or indirect routes via consumption of food, water, and infected items on the ground [2–4]. Biliary cirrhosis may also be a tardy sequel of intrabiliary perforation of liver hydatid cysts [5–7]. The majority of patients show an individual organ involvement with a single cyst, and 75–85% of cysts are localized in the liver of patients [2, 5]. Some cysts may grow at an average rate of 1–20 mm per year, and these patients survive with no evident changes for a long time; other cysts can become calcified and completely disappear [5, 8]. An enlarging cyst may compress and cause atrophy and fibrosis of the liver [5, 9]. The compression and displacement of biliary ducts can frequently produce a spontaneous rupture in biliary ducts. Timely detection and therapy are mandatory in the case of an intrabiliary perforation or rupture of a liver hydatid cyst, which can result in the obstruction of the biliary duct with 50% mortality [8–10].

Imaging tools, such as ultrasonography (U/S), abdominal computed tomography (CT), magnetic resonance cholangiopancreatography (MRCP), and endoscopic retrograde cholangiopancreatography (ERCP), are useful devices to diagnose the disease. U/S and CT scans are the first diagnostic tools of choice and can be applied under all conditions [1, 2]. Of the more invasive instruments, ERCP can help establish definitive detection and treatment with sphincterotomy in patients affected by intra-biliary rupture of a cyst, and MRCP can diagnose the site of the obstructions of the biliary system [6, 7, 10, 11]. This case report describes the clinical presentation of a patient with liver echinococcosis, the various complications of a hepatic hydatid cyst, especially its intrabiliary rupture, and the methods of diagnosing and managing cystic biliary fistulae.

## Case presentation

**Fig. 1:**
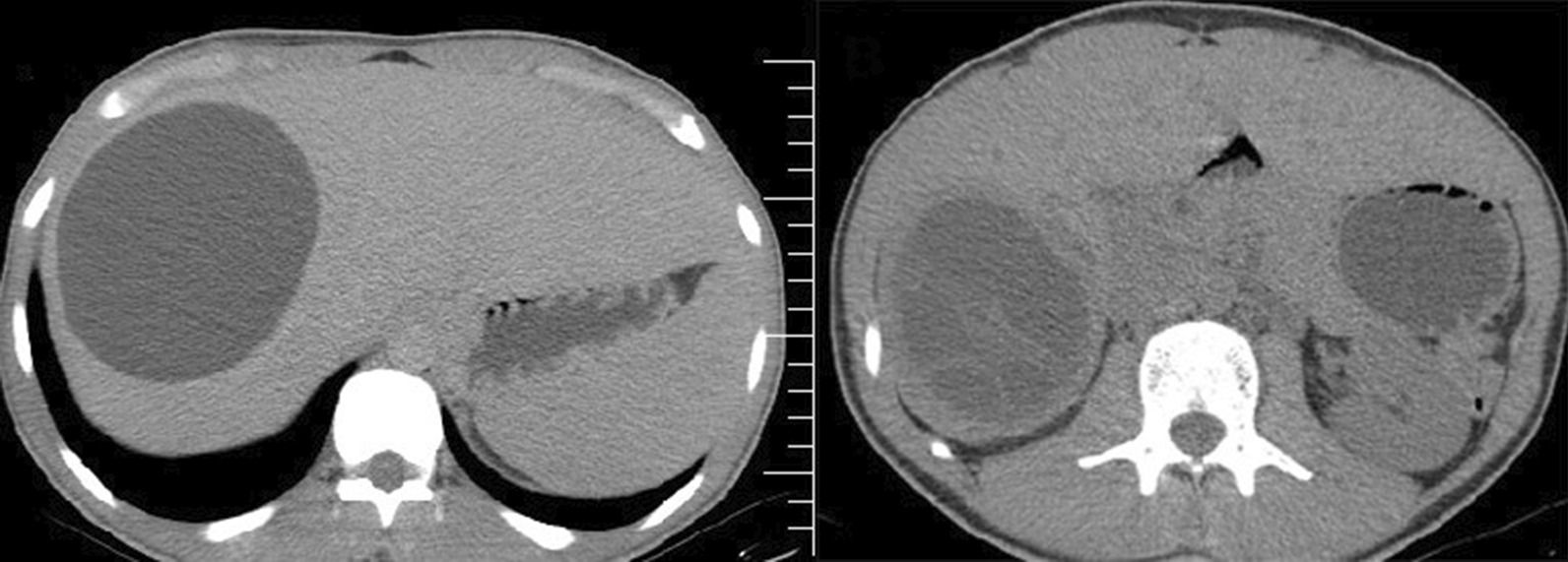
Computed tomography scan of the liver showing two cysts in segments II and VII, respectively, with septation, of which one was intact and the other had ruptured

A 21-year-old Iranian man presented to the emergency ward of our hospital with 5 days of illness and a history of right upper quadrant abdominal pain, fatigue, fever, icterus, vomiting, and no appetite. He was examined physically, and abdominal tenderness was detected in all four quadrants, and scleral icterus. His blood pressure was normal at 110.70 mmHg and oxygen saturation rate was of 95% on ambient air. Axillary temperature was 38.8 °C. His medical history showed that was being treated with albendasol 800 mg daily for months because of two hydatid cysts in the liver. One of the cysts was located on the dome of liver segment III and the second was on segment VII just over the right kidney (Fig. 1). On the CT scan of the liver, two cysts were observed in segment II and VII with septation, of which one was intact and the other one had ruptured. After 2 weeks on albendazole, fatigue, fever, icterus, and vomiting were present, which was the complication of albendazole since the values for all liver function tests had increased.

Laboratory findings revealed increased white blood count (WBC; 17,000 K/uL, reference value 4–10.8 K/uL) and elevated liver enzymes (aspartate transaminase [AST] 120 U/L [reference value 11–72 U/L]; alanine transaminase [ALT] 83 IU/L [reference value < 40 IU/L]; alkaline phosphatase [ALP] 1250 IU/L; total bilirubin 9 mg/dL [reference value < 1.2 mg/dL]). The direct and indirect bilirubin measurements were 4 and 5 mg/dL, respectively, C-reactive protein concentration was 11 U/mL (reference value 0–0.5 mg/dL, amylase 320), and lipase was 180 IU/L. A prothrombin time of 42 s (reference value 9.6–14.2 s), activated partial thromboplastin time of 48 s (reference value, 20–38 s), and international normalized ratio of 5 (reference value 0.85–1.2) were also detected in the tests. The remaining laboratory test results were within normal limits. These test results show hepatitis due to albendazole toxicity.

**Fig. 2:**
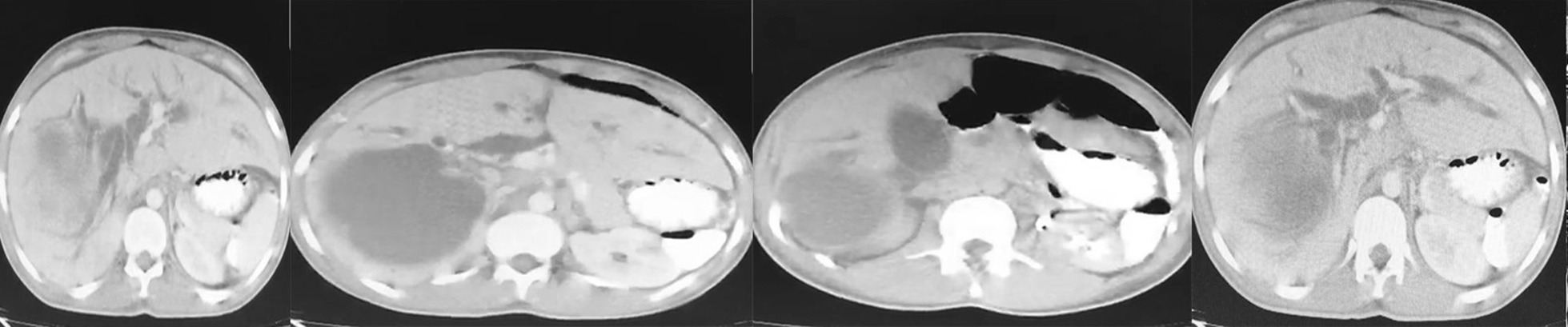
Axial contrast enhanced computed tomography scan showing unilocular hepatic cysts with detached laminated membranes compatible with hepatic hydatid cysts on segments II and IV. Intrahepatic biliary duct dilation is also seen

**Fig. 3:**
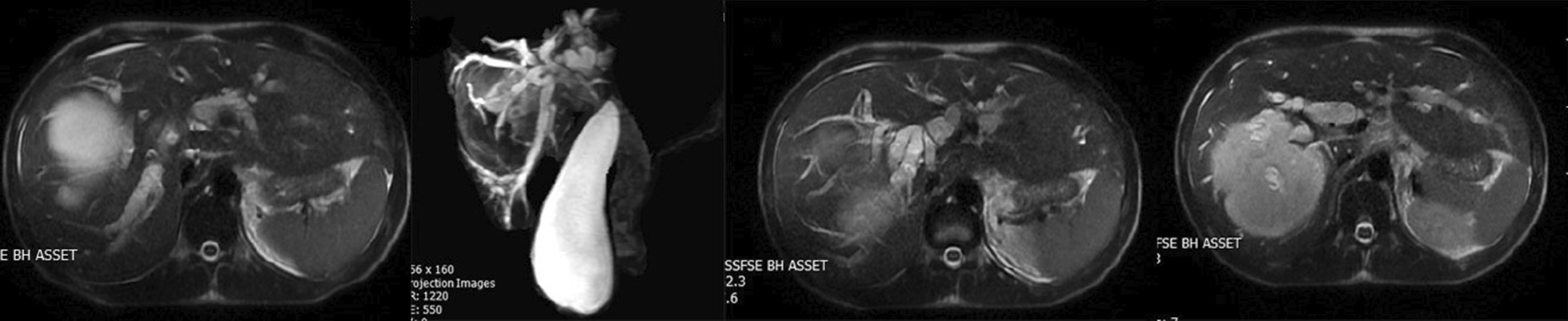
Magnetic resonance cholangiopancreatography shows cyst rupture into the common hydatid cyst membranes and hepatic duct in the common bile duct. A hydatid cyst was also observed on segment II. Laparotomy and common biliary duct exploration established that the bile duct was obstructed with hydatid membranes and daughter cysts

In addition to considering these test findings and the patient’s ailments, we also used abdominal U/S to arrive at the diagnosis. Abdominal U/S revealed intrahepatic and extrahepatic bile duct dilation; the gallbladder was dilated but had normal wall thickening. Large intact hepatic cysts were observed in segment IV and another one were in segment II with detached laminated membranes, possibly indicative of a ruptured or complicated liver cyst. An intravenous contrast CT scan was performed for more evaluation and revealed unilocular hepatic cysts with segregated laminated membranes that corresponded to hepatic hydatid cysts on segment II and other unilocular intact hepatic cysts on segment IV (Fig. 2). Further observations indicated intrahepatic and extrahepatic biliary duct dilation (Fig. 3).

The reason for bile duct dilatation was investigated by MRCP, which showed the rupture of cysts of segment II into the intrahepatic ducts, common hepatic duct, and common biliary duct (CBD). In addition, laminated membranes of the hydatid cyst and daughter cysts were found in the CBD, causing the obstruction (Fig. 3).

The patient underwent laparotomy due peritonitis resulting from the rupture of the liver hydatid cyst in the abdominal cavity with a right extensive subcostal incision. During exploration, 500 cc bile fluid was aspirated from the abdominal cavity and sent for analysis. The pancreas was inflamed, and its appearance showed pancreatitis. The liver was carefully inspected; one collapsed infected cystic lesion with a small perforation was present in segment II just over the right kidney.

Our approach for surgical treatment began with the aspiration and evacuation of the cyst contents. To explore for any purulent or bile contents, cystostomy and irrigation were performed. Also, fibrotic tissue around the cyst was resected and the site of bile leakage ligated with a non-absorbable suture. Capitonnage, omentoplasty, and insertion of a Foley catheter inside the incision were performed to remove the residual cyst and prevent recurrence. The gall bladder was highly dilated and was full of a white mucous fluid with obstruction of the cystic duct; choledocotomy and drainage of the common bile duct were performed with saline irrigation. Moreover, daughter cysts were removed from the common bile duct and intrabiliary ducts. At the end of the surgery, a T-tube was inserted in the site of choledocotomy and a corrugated drain was fixed. During exploration, another intact cyst was found in segment VII; this cyst was aspirated and the laminated membrane removed; the cavity was then irrigated with betadine 10%. Pricystectomy was performed, and a Foley catheter 18 was put into the remnant cavity and fixed to the abdominal wall. Finally, the CBD (diameter 30 mm) was examined; it was full of laminated membrane, daughter cysts, and debrides of the hydrated cyst. After extraction of all materials from the CBD and subsequent irrigation, a T-tube was placed in the CBD and fixed into place. A Penrose drain was placed in the abdominal cavity, and then the abdomen was closed.

**Fig. 4:**
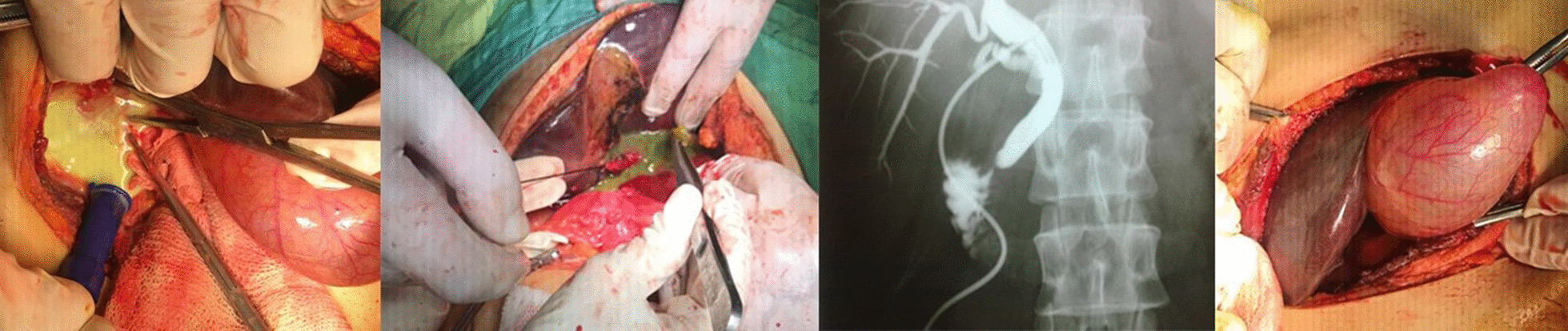
Pus from collected after exploration of common bilary duct and dilated gallbladder and T-tube cholangiography

Three days after surgery, the levels of bilirubin, amylase, lipase, AST, ALT, WBC, and ALP had decreased. The patient’s general condition (fever and appetite) was good, and he was discharged 10-day post-operation with good condition. Trans-T-tube cholangiography was performed 20 days after the operation with good results and was then removed (Figure 4)

## Discussion and conclusion

The complications of a liver hydatid cyst (LHC) are rupture into the biliary duct, abscess formation, and rupture into the pulmonary parenchyma and pleural space [1, 3–5]. Presentation of an intrabiliary rupture of LHC ranges from no symptoms in a minor perforation to obstructive jaundice, cholecystitis, cholangitis, pancreatitis, peritonitis, and/or septicemia [5, 6, 10]. In 10–37% of patients, the intrabiliary fistulae due to LHC are of the occult type, with 3–17% of patients having the frank type[5]. Rupture of the occult-type fistula rupture is typically asymptomatic and can be seen as a liver abscess or suppuration, or it can be present as a frank rupture [5, 8]. Clinically, observations are not specific at this phase [4, 5, 10]. Ruptured fragments of laminated membranes, daughter cysts, and other debridement of the cyst can penetrate the biliary tree and cause obstructive icterus, acute cholangitis, or, as in our case, septicemia [6, 10]. Additional presentations, such as acute pancreatitis, acute cholecystitis, and peritonitis due to hydatid material, have been discussed in published reports. Although fistulae between the biliary system and LHC are present in 80–90% of patients, the incidence of clinical presentation occurs only in 13–37% of patients [5, 8, 10]. The rate of huge intrabiliary rupture occurs in 5–17% of patients [5, 6, 8, 10]. Although in these conditions, mortality and morbidity are high [3, 5, 6, 10], diagnosis is difficult in occult or minor fistulae between the biliary system and LHC, as the indications and radiological pre-surgical observations are not remarkable [3, 5, 6]. Biliary leakage is the highly frequent consequence of liver hydatid cyst [5, 6, 9, 10]. The patient described here presented with frank intrabiliary rupture.

The preoperative detection of the consequences of LHC is difficult, but clinical demonstrations and findings of in vitro assays can be suggestive of intrabiliary rupture, as in our case. U/S and CT scans may indicate a minor or huge intrabiliary rupture in the majority of cases, and magnetic resonance imaging is another imaging tool that can facilitate diagnosis. MRCP can show daughter cysts, separation of the membranes, dilated biliary tree, and hydatid cyst material in the biliary system [6–9]. In our case, the cyst had ruptured into both the abdominal cavity and biliary tree. The surgical treatment of LHC includes hepatic resection and total cystostomy, conservative cyst evacuation, resection of fibrotic priciest layer, and capitonnage with external drainage [2, 5–7, 11]. Current approaches for the treatment of LHC are usually aspiration, cyst evacuation followed by, capitonnage with external drainage, or omentoplasty [1, 2, 8]. The aim of surgery for LHC is to remove all cyst elements and prevent the spread of cysts content via minimizing complications [2, 5]. Aspiration and cyst evacuation followed by external drainage is a relatively harmless and easy approach and is helpful in the treatment of uncomplicated LHC [5]. The main disadvantage of this approach is that adverse events occur at high frequency after the surgery, such as cyst-biliary fistulae, bilio-cutaneous fistulae, bilomas, and bile peritonitis (4–28%) in the remnant cavity for a long time [11–13]. In practice, we never perform hepatic resection [1, 2]. After surgery for LHC, external biliary fistulae are usually closed extemporaneously [5]. In a study involving 304 cases, external biliary fistulae closed extemporaneously within 2–4 months [13]. In another report, all fistulae closed extemporaneously within a maximal duration of 38 days; the prolonged fistulae drainage and morbidity weree high [14]. Medical treatment is usually indicated before surgery to sterilize the fistulae and to avoid recurrences. In medical treatment, disseminated hydatidosis has been indicated. To date, drugs of the benzimidazole family (albendazole or mebendazole) are used to treat hydatidosis, but albendazole is the best pharmacological option [14, 15]. In a few patients with post-surgical external biliary fistulae, it is generally accepted that endoscopic sphincterotomy, with or without stenting or naso-biliary drainage, mitigates the high intrabiliary pressure and improves timely closing of these fistulae even when the distal biliary obstruction is not present [11]. In our case, biliary drainage stopped 20 days post-operation, and the T-tube was removed.

Intrabiliary perforation of the LHC is a scarce event but one with severe consequences. Physicians treating patients referred to medical centers while complaining of abdominal pain, fever, peritonitis, decreased appetite, and icterus should recognize the possibility of perforation of LHC, as opposed to hydatid disease. Early diagnosis of complications and aggressive treatments, such as ERCP and surgery, are vital.

## Data Availability

Not applicable.

## Abbreviations

CBD: Common biliary duct
CT: Computed tomography
U/S: Ultrasonography
